# Analysis of the Continuous Feeding of Catalyst Particles during the Growth of Vertically Aligned Carbon Nanotubes by Aerosol-Assisted CCVD

**DOI:** 10.3390/nano12030449

**Published:** 2022-01-28

**Authors:** Celia Castro, Rodrigo Fernández-Pacheco, Mathieu Pinault, Odile Stephan, Cécile Reynaud, Martine Mayne-L’Hermite

**Affiliations:** 1 NIMBE, CEA, CNRS, Université Paris-Saclay, 91191 Gif sur Yvette, France; celia.castro@univ-rouen.fr (C.C.); mathieu.pinault@cea.fr (M.P.); cecile.reynaud@cea.fr (C.R.); 2 Groupe de Physique des Matériaux, Normandie University, UNIROUEN, INSA Rouen, CNRS UMR 6634, 76000 Rouen, France; 3 Laboratoire de Physique des Solides, Université Paris-Saclay, CNRS UMR 8502, 91405 Orsay, France; pacheco@unizar.es (R.F.-P.); odile.stephan@u-psud.fr (O.S.); 4 Laboratorio de Microscopías Avanzadas (LMA), Instituto de Nanociencia y Materiales de Aragón (INMA), CSIC-Universidad de Zaragoza, 50018 Zaragoza, Spain

**Keywords:** carbon nanotubes, aerosol assisted CCVD, ferrocene, cobaltocene, EELS, WDS

## Abstract

Aerosol-assisted catalytic chemical vapor deposition (AACCVD) is a powerful one-step process to produce vertically aligned carbon nanotubes (VACNTs), characterized by the continuous supply of the catalyst precursor (metallocene). The behavior of catalyst species all along the synthesis is essential for the continuous growth of VACNTs. It is there investigated through detailed observations and elemental analyses at scales of VACNT carpets and of individual CNTs. Our approach is based on two complementary experiments: quenching of the sample cooling, and sequential injection of two distinct metallocenes. Metal-based nanoparticles nucleated in the gas-phase during the whole synthesis duration are shown to diffuse in between the growing VACNTs from the top of the CNT carpet towards the substrate. They are much smaller than the catalyst particles formed on the substrate in the initial steps of the process and evidences are given that they continuously feed these catalyst particles at the VACNT roots. Particularly, the electron energy-loss spectroscopy (EELS) analyses of metal-based segments found into a single CNT show that the second injected metal is very gradually incorporated in the particle initially formed from the metal firstly injected. The feeding of the catalyst particles by the nanoparticles continuously nucleated in the gas-phase is therefore an essential feature of the base-growth of CNTs by AACCVD.

## 1. Introduction

Catalytic Chemical Vapor Deposition (CCVD) is to this day the most promising method for the synthesis of controlled carbon nanotubes (CNTs) for industrial production. The carbon precursor can be either gaseous or liquid, and the catalyst particles can be either pre-deposited on substrates [1,2,3] (two steps process) or formed during a one-step process from a metal precursor [4,5,6,7,8] injected simultaneously with the carbonaceous precursor. In both cases, the optimization of the process requires a deep understanding of the involved CNT growth mechanism. Among the one step techniques, the aerosol-assisted CCVD method consists in generating an aerosol in order to simultaneously inject both catalytic and carbon precursors (generally ferrocene in toluene) in the reactor. This technique leads to a continuous and fast growth of clean carpets of vertically aligned multiwalled carbon nanotubes (VACNTs) [6]. These macroscopic VACNT carpets are synthesized through a base-growth mechanism from catalyst particles formed on the surface of the substrate in the early stages of the process [9,10]. It had been shown that, as soon as the temperature is high enough to induce the thermal decomposition of the metallocene precursor, the partial vapor pressure of iron is high enough for a homogeneous nucleation of small metal-based nanoparticles (NPs) to occur in the gas phase. These are deposited on the substrate along the reactor leading to the formation of the catalytic particles by coalescence [11]. Evidence has been given for the diffusion of carbon species through the growing CNT carpet down to these catalytic particles all along the synthesis duration [9]. Moreover, the growth rate of CNTs was shown to be constant in a wide range of synthesis conditions [9,12,13,14,15,16]. However, in the absence of supply of catalyst precursor, the growth of CNTs is stopped [9] suggesting that the continuous refreshment of the catalytic particles prevents their deactivation. In addition, the CNTs growth starts as soon as the catalyst precursor is injected as shown through in situ XRD analysis during the formation of VACNT carpets [17]. The continuous refreshment induces the encapsulation of metal-based species inside the CNT central cores, thus forming elongated particles or nanowires of different nature [17,18,19]. The encapsulation is driven by their mobility at high temperature (800 °C) and capillarity forces inside the growing CNT [17]. This renewal is effective throughout the growth process, as demonstrated by specific syntheses using different metallocene precursors in a sequential manner, implying that the metal precursors are able to diffuse through the growing carpet to the substrate in the same way of the carbon precursors [19,20]. Therefore, the continuous supply of catalytic precursor is an essential feature of this one-step CCVD process for the growth of VACNTs.

In this context, our objective is to go deeper in the understanding of the metal-based species behavior in aerosol-assisted CCVD process, specifically the nanoparticles nucleated in the gas phase during the entire process [11]. The aim is to characterize how these nanoparticles progress within the growing carpet to interact with the active catalytic particles. Our experimental approach is focused on the elemental analysis of metals along the VACNT carpet cross-section using an electron microprobe based on Wavelength Dispersive X-ray Spectrometry (WDS) and along isolated CNTs core by spatially resolved EELS analysis [21]. Two main adjustments of the process are used. First, the standard cooling procedure of the reactor at the end of the synthesis is replaced by a quenching procedure in order to observe the sample in a state as close as possible to the real state during the synthesis [18]. Secondly, a sequential injection procedure, which is already used to study the carbon species progress [9], is implemented, cobaltocene replacing ferrocene in the second sequence.

In the following, we first investigate the distribution of the catalyst species in the whole VACNT carpet through the quenching of the synthesis. Then, the sequential synthesis with different metallocene precursors is detailed before analyzing the resulting bilayer. Finally, we describe the elemental analysis of the metal particles located inside a single nanotube from the catalyst particle from its root towards its top, leading us to the discussion of the progression of the catalytic species during the VACNT growth.

## 2. Materials and Methods

The synthesis setup and standard procedure have been described previously [6,22,23]. Briefly, the precursor mixture is made of ferrocene or cobaltocene (Acros Organics, part of Thermofisher Scientific, Illkirch, France) dissolved in toluene (99.9%, Analytical grade, Merck, Sigma-Aldrich Chemistry products, St. Quentin Fallavier, France), the resulting solution is nebulized through an aerosol generator, and the aerosol obtained is carried by an argon flow (without any enhancer) to the reactor through an evaporator at 200 °C. The cobaltocene is very sensitive to oxidation in air and to hydrolysis in solution. It is, therefore, necessary to use a specific protocol of dissolution in anhydrous toluene under an inert atmosphere (Ar) into a glove box. In addition, the container of the injector is directly filled in the glove box. The aerosol pyrolysis takes place in a quartz reactor at atmospheric pressure located in a 45 cm long tubular furnace. The furnace has a parabolic-like temperature profile [11] with an isothermal area (±10 °C) that ranges from 18 to 27 cm for a 800 °C imposed temperature or from 20 to 27 cm for 850 °C. VACNT growth occurs on the reactor walls and on substrates (quartz, silicon) previously placed into the reactor.

The furnace has been fixed on wheels [18], allowing its horizontal translation along the fixed quartz reactor. By moving the furnace, the temperature of the substrates at a given position in the reactor can be changed. The furnace can be either shifted just a few centimeters away in order to adjust the temperature of the samples along the temperature gradient or completely moved in order to suddenly place the reactor out of the heating area, thus performing the samples quenching down to room temperature. This procedure has been previously described in details [18].

**Figure 1:**
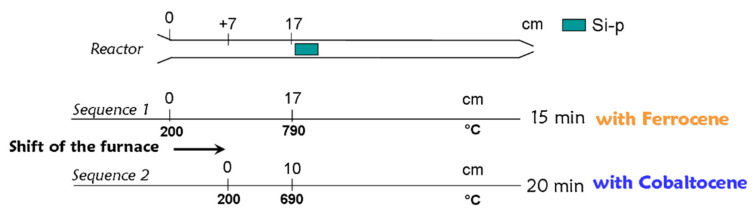
Chronology of sequences used in the bilayer synthesis with indication of substrate position in the furnace and duration for each injection sequence.

In order to perform sequential synthesis with two different precursor mixtures, the set-up is equipped with two tanks connected to the injection system. The double injection experimental procedures are similar to those described by Charon et al. [23]. Two different metallocene precursors are used sequentially. A solution with 2.5 wt% of ferrocene in toluene is poured in tank A while 1.25 wt% of cobaltocene in anhydrous toluene is in tank B. Preliminary experiments have been performed to adjust the experimental procedure while using cobaltocene [20], since it demonstrated a weaker thermal and chemical stability than ferrocene [24,25,26,27]. At a temperature of 800 °C, the VACNT optimal growth conditions are obtained at the entrance of the furnace when using cobaltocene and at the center for ferrocene. Therefore, the furnace is quickly moved (few seconds) by 7 cm upstream when switching from tank A to tank B (Figure 1). First the solution with ferrocene is injected for 15 min and then the solution with cobaltocene for 20 min. Those durations are necessary in order to obtain a CNT carpets thick enough to allow observations and analyses, since the growth rate is weaker with cobaltocene than with ferrocene.

All samples scratched from the reactor or grown on substrates are analyzed with different techniques. Morphology and thickness of CNT carpets are investigated by scanning electron microscopy (SEM Leo Gemini1525, field emission gun, Zeiss France, Rueil Malmaison, France). CNTs individually dispersed in absolute ethanol and deposited on a grid are characterized by transmission electron microscopy (TEM Philips CM 12, Philips, Eindhoven, The Netherlands). The elemental analysis profile is performed on carpet cross-section using a Cameca SX 50 electron microprobe (Gennevilliers, France) based on Wavelength Dispersive X-ray Spectrometry (WDS). Raman spectroscopy on CNT carpet was performed on a Renishaw Invia Reflex microspectrometer (Marne La Vallée, France). The laser beam of 3 µm in diameter was a doubled Nd: YAG with 532 nm in wavelength. Spatially resolved chemical analyses are carried out on individual CNTs in a scanning transmission electron microscope (STEM) VG HB 501 (VG Microscopes Ltd., East Grinstead, UK) equipped with a field emission gun operated at 100 kV and fitted with a Gatan 666 spectrometer, optically coupled to a CCD camera. Spatially resolved EELS analysis [21] is employed to investigate the content of the relevant chemical elements in metal particles and nanowires filling the CNTs. The convergence and the collection angles are, respectively, 15 mrad and 24 mrad.

## 3. Results

### 3.1. Iron Content Distribution along the VACNTs

**Figure 2:**
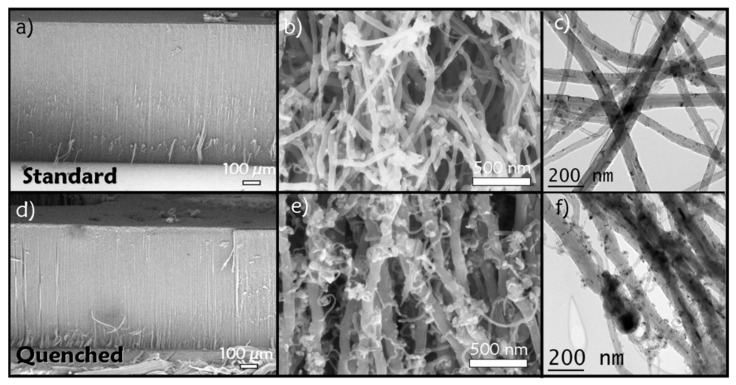
(**a**,**b**,**d**,**e**) SEM images and (**c**,**f**) TEM images of CNTs grown from toluene and ferrocene aerosol mixture with the standard (**a**–**c**) and quenched cooling down procedure (**d**–**f**). (**a**,**d**) corresponding to the cross-section of the CNT carpet, (**b**,**e**) to the higher magnification in the top part of the cross-section of the carpet, (**c**,**f**) to CNTs dispersed on TEM grids.

The distribution of iron along VACNTs is studied from samples synthesized from a solution with 2.5 wt% of ferrocene in toluene. Two synthesis that differ only in the cooling procedure are performed at 850 °C during 15 min. The first sample is cooled down according to the standard procedure, where the cooling is governed by the thermal inertia of the furnace and taking about two hours. The second one is cooled down in a few minutes through the quenching procedure by quickly moving the furnace as described above [18]. SEM images of the samples (Figure 2a,d) show carpets made of VACNTs, as generally obtained by this synthesis method. The first observation is that the carpet originated from the quenching procedure (Figure 2d) is thinner (790 µm) than the carpet obtained from the standard cooling (925 µm, Figure 2a). The (b) and (e) panels of Figure 2b,e show the top part of the cross-section of the samples, which corresponds to the early stages of the CNT growth and is therefore the more disordered part of the carpet [10]. For the quenched sample (Figure 2e) numerous nanoparticles can be observed outside the nanotubes as well as small and thin CNTs grown on the aligned nanotubes of the carpet, which are not present in the standard sample (Figure 2b). In the middle of the carpet the same observation can be done with more particles outside the nanotubes in the quenched sample (not shown here). This result is confirmed by TEM observations presented on Figure 2c,f. The size of these NPs is much smaller than the diameter of the VACNTs.

**Figure 3:**
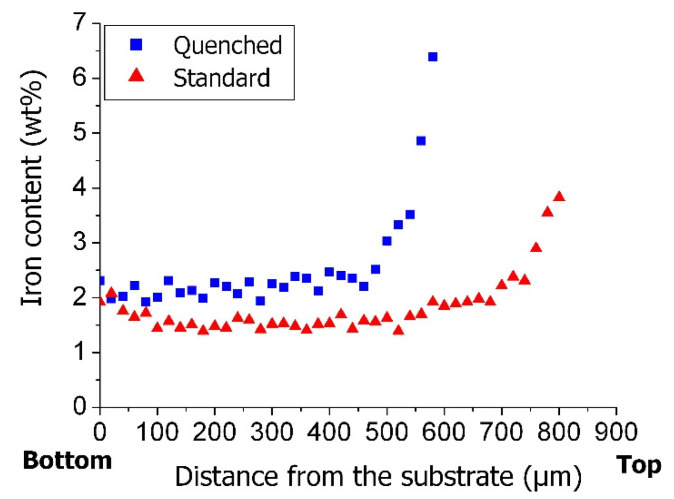
WDS profiles of iron content along the cross-section of VACNT carpets from the top to the base (substrates) for samples coming from (■) quenched and (▲) standard cooling procedures, respectively.

The iron content measured by WDS (Figure 3) all along the carpets is, in both cases, maximum at their top and decreases quickly (along a thickness of almost 100 µm) to reach a value that remains nearly constant down to the base of the carpet. However, for the quenched sample, the iron content is much higher at the carpet top (6.5 wt% as compared to 3.7 wt% the not quenched sample) and it remains also the highest along the carpet (between 2 and 2.5 % for the quenched sample as compared to 1.5 wt% for the not quenched sample). At the carpet base, the iron content is in both cases close to 2 wt%.

### 3.2. Results of the Sequential Synthesis: Bilayer Formation

**Figure 4:**
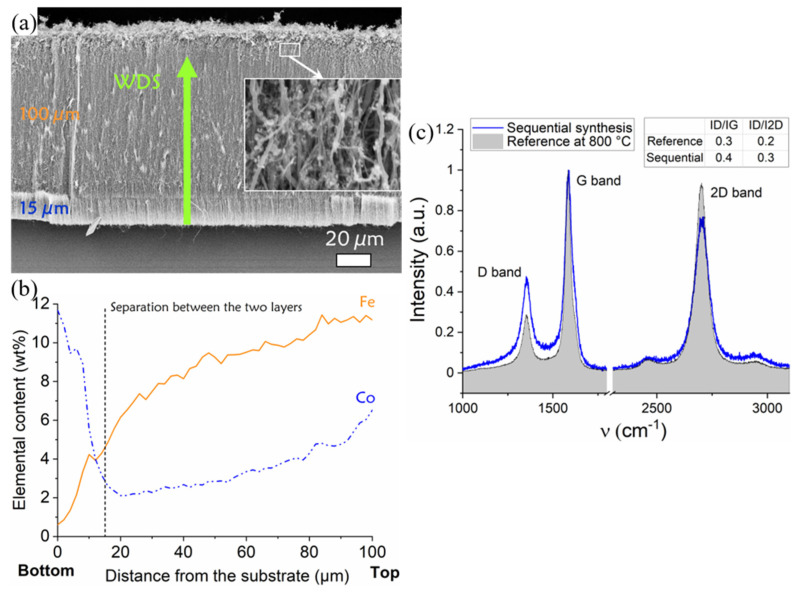
(**a**) SEM image of the bilayer carpet obtained sequentially from ferrocene and cobaltocene metallic precursors. (**b**) WDS elementary profile of iron and cobalt (wt%) performed along the cross-section of the CNT carpet from the substrate. The vertical dashed line indicates the transition zone between the two VACNT layers defined by SEM observations. (**c**) Raman spectra measured on the bilayer carpet compared to a reference sample synthetized with FeCp2 at 800 °C.

SEM observations show a carpet composed of two distinct layers with a separation line clearly distinguishable at 15 µm above the substrate (Figure 4a). Both layers exhibit a good CNTs alignment. The thicknesses are 100 µm for the top layer and 15 µm for the bottom one. The carpet thickness is lower than that usually obtained at 850 °C presented in part 3.1, which is consistent with the lower synthesis temperature of 800 °C used here [10]. Concerning the top of the carpet, the inset in Figure 4a revealed a disordered CNT area and many metallic particles.

The graphitization quality of the CNTs has been controlled by Raman spectroscopy (Figure 4c). Raman spectra measured on the bilayer carpet is compared to a reference CNTs carpet synthetized with FeCp2 at 800 °C. Both spectra present strong 2D harmonic bands related to perfect graphitic structure of carbon, confirming the presence of well graphitized CNTs. The intensity ratios of D to G and D to 2D bands are presented in Figure 4c. Measurements have been repeated twice on each sample. For both ratios, we observe a slight increase for the bilayer carpet. The graphitization quality is then slightly degraded in the bilayer carpet, but still in the range of very good quality CNTs.

Elemental analysis by WDS along the cross-section of the bilayer carpet shows that iron is mainly found in the upper layer (Figure 4b). Iron content reaches its maximum value at the top of the carpet and decreases relatively slowly along the layer. This behavior is different from the one observed in the iron profiles in carpets growing from a unique sequence (Figure 3) where the decrease is sharper. Cobalt is mainly located in the bottom layer, with a pronounced maximum content near the substrate at the base of the carpet. The cobalt content then decreases quickly as the distance from the substrate increases and until 20 µm, position for which it reaches its minimum value around 2 wt%. Beyond this position, the cobalt content increases slowly to reach a value around 6.5 wt% on top of the carpet in the upper layer. Interestingly, the profiles of the two elements intersect at 12 µm above the substrate, which is very close to the separation line between the two layers located at 15 µm above the substrate, as observed by SEM. At the limit between the two layers, the iron content is still significant (about 4 wt%) and decreases down to 1 wt% near the substrate.

**Figure 5:**
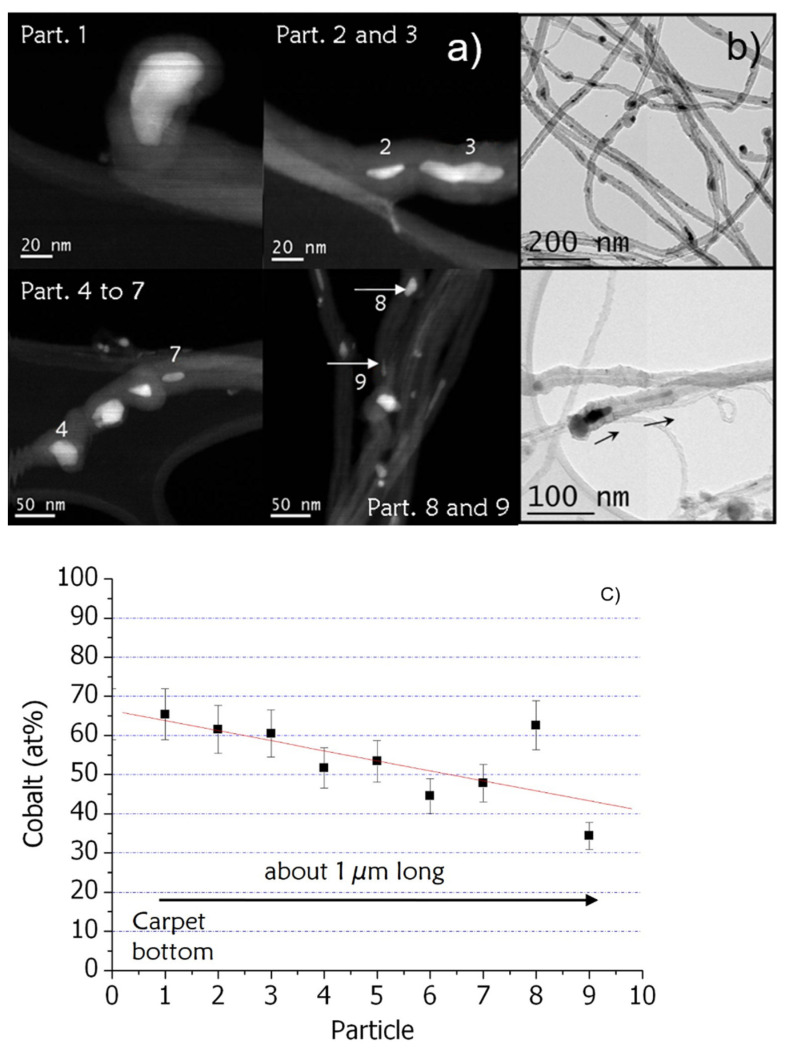
(**a**) annular dark field STEM images of analyzed particles by EELS along the bottom part of one CNT. (**b**) TEM images of CNTs obtained in the sequenced synthesis, (**c**) evolution of the cobalt content as compared to the iron one as a function of the analyzed particles (Co/(Fe + Co)). The first particle corresponds to the catalyst particle located at the base (root) of the CNT and the last particle corresponding to the upper particle identified.

STEM and TEM observations (Figure 5a,b) on individual CNTs (previously dispersed and deposited on TEM grids) show both particles and nanowires encapsulated in the hollow core of CNTs. STEM-EELS local analyses (Figure 5a,c) performed along individual CNTs from the catalyst particle at its base to the following encapsulated nanowires allow us to determine how both elements are locally distributed and to evaluate the possible presence of alloys. Different configurations are observed: nanowires and particles containing only one of the two elements, either iron or cobalt, or a mixture of both [19,20]. Moreover, extracting from the EELS spectra the signal of each element gives a quantitative estimation of the concentration of that element [28]. A detailed analysis is conducted within a single CNT starting from the particle at the base of the CNT, which is supposed to be the catalyst particle initially located at the substrate surface and continuing with the successive segments found inside this CNT going towards its top. We may observe that the absolute accuracy in iron and cobalt contents is in the order of 10 at% because we did not performed calibration from a known reference sample. However, as the thickness of the different analyzed particles is comparable, the accuracy in the relative composition is in the order of 1%. The case presented in Figure 5c is typical of single CNT with particles containing a mixture of iron and cobalt. The cobalt content expressed as the Co/(Fe + Co) atomic content is relatively high in average, around 50 at%. It is maximum for the catalyst particle located at the base of the CNT and decreases progressively in the nanowires along the inside of the CNT considered (Figure 5c). This result confirms the WDS analysis along the section and clearly shows a depletion of the cobalt amount in the catalytic particles and along the nanowires up to the limit area between the two layers.

## 4. Discussion

Regarding first the iron distribution in a CNT carpet, the elemental profiles along carpet cross-sections obtained by WDS (Figure 3) show an increase of iron content in quenched samples in comparison with the standard samples, but the height of standard samples is always higher than the quenched ones. Interestingly, the integral of both curves has almost the same value. The main difference in terms of iron content seems to come from the number of metal-based nanoparticles observed outside the CNTs by SEM/TEM (Figure 2e,f), which is significantly higher in the quenched sample. The size of these NPs is about six times lower than the diameter of both nanotubes and catalytic particles (Figure 2e,f) and is consistent with what is expected for the size of NPs nucleated in the gas phase. In the quenched sample, the NPs are observed frozen at their place during the synthesis and appear distributed along the CNTs, in agreement with a continuous diffusion through the carpet all along the CNTs towards the substrate during the growth process. An essential feature is the absence of any iron accumulation at the carpet base as demonstrated by the constant iron content in the WDS profile over a long distance from the substrate. This indicates that the diffusing NPs must be consumed as they reach the substrate level. In the case of standard cooling, the nanoparticles already formed at the synthesis stop can continue to progress towards the base of the carpet as long as the temperature is high enough for their diffusion or mobility to go on, and therefore they are much less present outside the CNTs at the end of the cooling step. This explains the difference in carpet thicknesses, since the growth activity is stopped by the quenching, leading to a smaller carpet [18], while in the standard cooling, the NPs, still progressing, can feed the catalyst particles at the CNT roots and promote the CNT growth as long as carbon precursors are available.

Regarding now the sequential synthesis where the metallic precursor is changed from ferrocene to cobaltocene, results show that the upper layer is formed by the first injection sequence with ferrocene and the lower layer by the second one with cobaltocene in agreement with the base growth mechanism [9], even when the nature of the catalyst is changed [19]. The top layer formed with ferrocene contains a significant amount of cobalt that increases towards the upper surface of the carpet. This can be explained by the diffusion, towards the substrate, of the cobalt NPs formed in the gas phase, even if part of them can be trapped inside the carpet, in between the CNTs. In the bottom layer formed with cobaltocene, cobalt content is the highest, but a small amount of iron is still present. In addition, local analysis by EELS (Figure 5) shows that the catalytic particle at the base of the CNT is richer in cobalt than nanowires encapsulated inside CNTs and that this enrichment in cobalt is very gradual and even incomplete. This slow enrichment is compatible with the incorporation of small NPs into a much larger catalyst particle.

**Figure 6:**
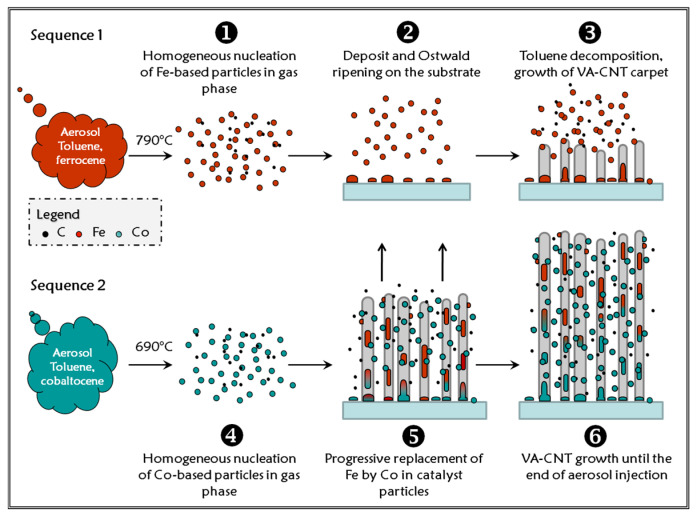
Diagram summarizing CNT carpet growth from sequential injection process including a first sequence using ferrocene followed with a second sequence using cobaltocene.

Finally, the WDS profiles of the VACNT carpets can be better understood considering that the metal content result from two main components, as illustrated in Figure 6. The first component is due to the metal-based NPs nucleated in the gas phase and diffusing through the carpet: part of these NPs can be trapped in between the CNTs, particularly at the top of the carpet, and contribute to the WDS profile. The second component of the WDS profile is due to the encapsulation phenomena induced by the renewal of the catalyst particles by the fraction of NPs that reach the substrate. The encapsulated segments are distributed all along the inner channels of the growing CNTs, suggesting that they are the main part of the metal content in the carpet.

## 5. Conclusions

Global as well as local characterizations have been combined in order to achieve a detailed analysis of the metal-based species in VACNT carpet grown by aerosol-assisted CCVD. The two synthesis approaches developed complementary answer to the dynamic steps followed by the catalyst species during the VACNTs growth. By quenching the cooling of the synthesis, it was possible to identify small metal-based nanoparticles in between the growing CNTs and to show that they are able to diffuse to the substrate where they are not accumulating but are consumed for the continuous CNTs growth. These NPs are reminiscent of the nanoparticles nucleated in the gas phase from metallocene supplied continuously by the aerosol. Through a sequential synthesis first using ferrocene, then cobaltocene as catalyst precursors we demonstrated by EELS analysis that the existing iron-based catalyst particles are very gradually enriched by newly injected cobalt-based species. This slow refreshment suggests that the small NPs formed in the gas phase and diffusing towards the carpet are incorporated in the catalyst particles and then play an essential role not only in their formation at the beginning of the synthesis, but also in their renewal all along the continuous growth of the VACNT carpets. Such important results regarding the progress of catalyst-precursor nanoparticles in a one-step CCVD process and obtained through SEM and TEM analysis opens towards complementary studies at the atomic scale to understand in more detail the behavior of catalysts.

## Data Availability

The data presented in this work are available upon request from the corresponding author.
